# Crowdsourced cycling data applications to estimate noise pollution exposure during urban cycling

**DOI:** 10.1016/j.heliyon.2024.e27918

**Published:** 2024-03-17

**Authors:** Rebecca Wogan, John Kennedy

**Affiliations:** Department of Mechanical, Manufacturing and Biomedical Engineering, Trinity College Dublin, University of Dublin, D02 PN40 Dublin, Ireland

**Keywords:** Noise pollution exposure, Cyclist experiences, Cyclist behaviors, Strava, Environmental health data

## Abstract

This research demonstrates a methodology to integrate freely available datasets to understand the relationship between road traffic noise and cycling experiences in a medium sized city. An illustrative example of the methodology was drawn from data for Dublin, Ireland. We aggregate local environmental data with 81,403 Strava cycle trips, contextualised by feedback from 335 cyclists to estimate exposure levels and infer impacts on experiences and behaviours. Results demonstrate that cyclists recognise that they are subjected to increased noise levels and experience negative psychophysical consequences as a result, but they tend to downplay the impact of noise as merely a minor annoyance. Noise also impacts behaviour, most noticeably through temporal and spatial detours. Geospatial mapping was used to visualise the relationship between noise pollution and cycling activity. Estimating traffic noise levels across two cycle routes, direct vs popular detour, revealed a +10 dB(A) increase in exposure for a saving of approximately 4 min on the direct route compared to the detour. Spatial inequities in exposure levels may have serious health consequences for cyclists in a city such as Dublin. The methodology is demonstrated as suitable for policy level interventions and planning purposes.

## 1 Introduction

An essential aspect of urban life is mobility [1], and with one-third of the world's population expected to reside in cities by 2030 [2], decisions regarding how we move in cities are pressing. Cycling is the most energy-efficient mode of transport, with unmistakable individual and societal health and well-being benefits due to physical exercise, space efficiency, no production of greenhouse gas emissions, air or noise pollution [3]. In contrast, motorised vehicles exert grossly negative consequences on society, including but not limited to traffic congestion, severe accidents, air and noise pollution [4]. Transitioning from driving a car to cycling can amount to a 9-fold benefit to the health of an individual [5], in the Irish context active travel saves the Health Service €29.2 m every year [6]. Importantly, greater numbers of people cycling and walking bring greater safety and enjoyment, unlike vehicular transport [7]. Not surprisingly, there are renewed drives towards increasing the numbers of people choosing to cycle as urban transportation [8].

However, a risk factor to achieving this modal shift may be noise pollution, of which motorised road traffic is the most severe source in cities [9]. The World Health Organisation recommends a limit of 53 dB L_den_ average exposure, with levels exceeding this associated with harmful health effects. Others argue that these WHO limits underestimate the detriments to health and wellbeing that can result from much lower levels [10]. In contrast to other stressors (such as exposures to second-hand smoke) which are falling, noise exposure is rising in Europe [11]. With traffic noise frequently exceeding 60 dB (dBA), exposure levels are highest among active travel users due to their proximity to traffic and lack of protection [12,13].

The detrimental and far-reaching impacts of noise on public health are becoming increasingly hard to ignore [14]. While researchers have long been aware of the dangers of noise induced hearing loss, more recently it has been shown that elevated or prolonged exposure can result in serious psychological and physiological impacts [15]. Physiologically, this can involve the release of stress hormones, arousal of the endocrine and autonomic nervous system, increased blood pressure, and heart rate fluctuations [16,17]. As a result people experience feelings of irritability, anxiety, nervousness, and mood swings [18]. Far from being a mere irritant, traffic noise is independently associated with increased morbidity and mortality risks including obesity [19], immune system dysfunction [20], cognitive impairment [21], attention disorders in children [22], and diabetes [23] among a myriad of others [10]. According to one survey, 44% of Europeans recognise the large impact noise exerts on people's health. However, Ireland featured the lowest recognition rate of any country at 16%, indicating that Irish citizens are significantly less informed than their EU peers on this matter [24].

While cycling facilitates building cardiovascular fitness, mental health, and improvements to quality of life [25,26], average noise levels cyclists experience have reportedly reached 71–75 dB, effectively acting as a “barrier effect”, harming and further deterring people from riding bicycles [13]. Research into cyclist noise exposure has begun to gain ground but is far from mature or mainstream. The majority of research on this subject has been published since 2015, suggesting an increase in concern for environmental factors related to cycling perhaps due to renewed global drives to increase the modal share [8] or advances in the development of low-cost, increasingly smart mobile technologies in the past decade [27]. Indeed, many studies have used portable noise sensors, for example [14,28,29], carried by research staff of the relative projects on predefined routes [12,13,29,30]. This reflects the frequent objective of simply monitoring city-level noise pollution rather than examining the experiences of people who choose to cycle. Indeed, fewer studies have been concerned with measuring the exposure of those who choose active mobility [18,[31], [32], [33]]. However, a recurring finding is that objective noise levels do not predict human perception of noise and reactions such as annoyance and stress [13,14,34].

Gössling et al. [18] highlighted several coping behaviours used by cyclists, demonstrating that the majority of cyclists detoured from the most direct path to avoid heavy traffic situations, increasing distances travelled by an average of 6.4%, at an extra cost of €0.24/km per person. Despite time and monetary costs, cyclists may substantially reduce their noise exposure taking these different routes [35]. To develop healthy, sustainable, and inclusive cities, both objective and subjective measures of environmental noise must be considered in development of cycle networks. Urban planners and transportation engineers alike need to be informed by cyclists' views, in other words, the end-users [36].

Crowdsourced and open-access initiatives can assist in identifying city streets that cyclists use or avoid, thereby uncovering those they deem to be unsafe or unpleasant, while further matching of environmental data like noise heatmaps could clarify why specific routes are or are not chosen. In this way active travel users can provide insight, participate in and inform decision-making [37]. Further, as people often report ignoring or even failing to perceive environmental risks [38], examining cyclists’ behaviours could provide an alternative way of measuring experiences of noise and urban cycling.

One popular fitness tracking smartphone application is Strava. Cyclist records from Strava have been widely used, for example, to understand where and how people cycle in cities [39], the barriers determining the gender gap in cycling [40], spatial and temporal variations in cycling volumes [41], cyclist interactions with new developments [42,43], as well as air pollution exposure during cycling [44]. One Canadian study monitored noise exposure of cyclists on pre-defined routes, however, the authors used Strava solely to validate cyclists’ GPS locations rather than to inform any insights regarding impacts of noise on cyclists [45]. Additionally, Strava data is utilised by a number of transportation bodies across the world [46,47]. The work will utilise such crowdsourced data to obtain insight into behavioural practices in relation to environmental health risks such as noise.

We explore the potential of integrating multiple sources of data to understand the relationship between road traffic noise and cycling experiences. Firstly, there is a lack of knowledge of the problem of cyclist noise exposure, particularly in Ireland and, therefore, it is currently difficult to incorporate noise considerations into transport policies and developments to assure gains in health and wellbeing. Secondly, in general, there is little research into the relationship between cyclist exposure and their behaviours. This research aims to develop a methodology to gain insight into real behaviour and subsequently inform data-driven policy. One way for city planners and policy makers to incorporate cycling into their planning process is by utilising a straightforward and inexpensive method. Herein we demonstrate one such feasible methodology, prior to any rigorous implementation of the technique. This methodology will answer the following questions.

1. Do cyclists report that noise impacts their experiences cycling in Dublin?
2. Is there evidence of impacts of noise on their behaviour?
3. Is there also evidence of noise impacts on behaviours in the crowdsourced data?
4. Can these datasets be used to estimate the exposure of an urban cyclist to noise?

## 2 Materials and methods

**Fig. 1:**
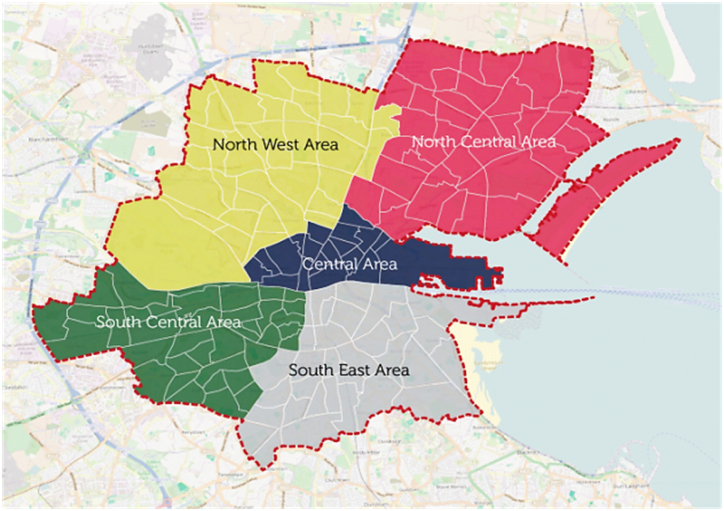
An overview of the region of interest featuring the five local administrative areas of Dublin City Council [51].

The illustrative study location is Dublin City, the capital city of Ireland, with a population of 588,233 [48]. The entire Dublin Metropolitan Area hosts a reported 95 km of “traffic-free” cycle routes and 118 km lanes physically separated from traffic [6]. Dublin has a cycling modal share of 6% as of 2019 [49] compared to 10–30% in many other cities [18]. Every day the people walking and cycling in Dublin remove up to 330,000 cars off the roads. The yearly individual and societal benefits of these behaviours include 3207 serious health conditions avoided, €1.1 billion generated for the economy, and reductions in greenhouse gas emissions equivalent to 340,000 flights from Dublin to Heathrow [6]. This study specifically considered the inner aspect of the city composed of the 5 local administrative areas of Dublin City Council (see Fig. 1) [50].

### 2.1 Data collection

**Fig. 2:**
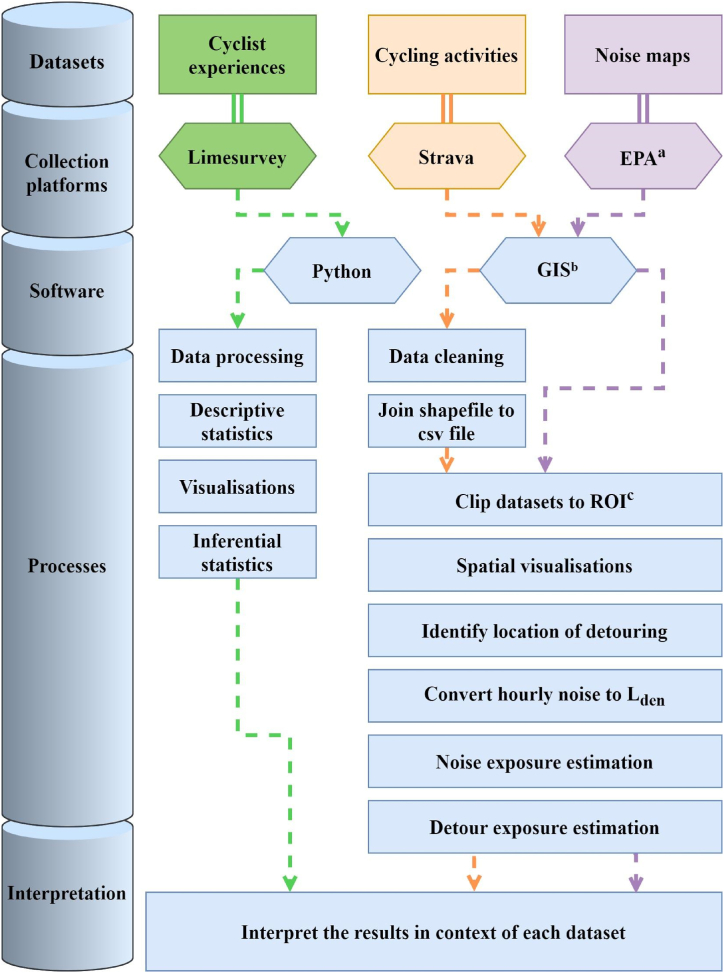
Data collection and analysis pipeline. ^a^ EPA, Environmental Protection Agency; ^b^ GIS, Geographical Information Systems; ^c^ ROI, Region of interest.

At present, monitoring the modal share of cycling in Dublin involves conducting manual counts of individuals commuting towards the city center at specific locations along the canal cordon, on certain dates throughout the year [52]. Several datasets and procedures were used herein including crowdsourced cycling data, municipal noise data, as well as one of the largest surveys conducted on cyclists in Dublin, all described below. These were synthesised to gain insights into the sound environment, while the surveys operated as a check of the validity of cyclists’ current experiences cycling in Dublin. See Fig. 2 for a visualisation of the data collection sources and analysis pipeline.

#### 2.1.1 Cyclist experiences

This project, received ethical approval from the relevant Trinity College Dublin ethics committee prior to commencement. Drawing on previous research, survey questions were drafted enquiring into participants' general experiences cycling in Dublin city, perceptions of traffic noise, and potential impact of noise on their behaviour [18,34]. In total, 19 questions were composed across 5 categories, for an outline see Appendix A TableA1. Six questions asked about (a) participants’ general cycling habits, the length and breadth of their participation in cycling. Five questions asked about (b) their experience of cycling in Dublin, including the perceived affective quality of the soundscape of Dublin streets. Four questions enquired about (c) cycling behaviours, three about (d) general demographics regarding age, gender, and life situation. One item consisted of (e) a noise sensitivity assessment.

**Table 1:** Summary of cyclist demographics obtained from the surveys.

Demographics	N (%)	
Gender rowhead		
Men		219 (65%)
Women		113 (34%)
Non-binary		3 (1%)
**Age** rowhead		
18–25		24 (7.2%)
26–35		106 (31.8%)
36–45		117 (35.1%)
46–55		70 (21%)
56–65		11 (3.3%)
66+		5 (1.5%)
**Life Status** rowhead		
Employed		295 (88%)
Non-waged work		35 (2.1%)
Unemployed		8 (2.4%)
Student		7 (7.5%)
**Experience cycling in Dublin** rowhead		
+3 years		252 (75.2%)
1–3 years		58 (17.3%)
3 months-1 year		19 (5.7%)
Less than 3 months		6 (1.8%)
**Frequency cycling** rowhead		
Every day		95 (28.4%)
Several times a week		184 (54.9%)
About once a week		22 (6.6%)
Several times a month		25 (7.5%)
Less than once a month		9 (2.7%)

The aforementioned noise sensitivity assessment included items of a reduced Weinstein Noise Sensitivity Scale (WNSS) [53], as previous research has shown that a limited number of items can still accurately define profiles of user's noise sensitivity [54]. Noise sensitivity refers to an individual's degree of aversion or reactivity to noisy situations [54]. People with high noise sensitivity pay more attention to noise, find it more threatening, and show slower reactions to noise [55]. High sensitivity has many adverse effects including elevated heart rate [56], greater annoyance [57], and increased risk of psychological ill-health when in noisy road traffic environments [58]. Sensitivity profiles can be used to predict who is more likely to experience negative effects on their health from noise, as well as who stands to benefit most from efforts to reduce their exposure [54].

Participants were recruited to complete the survey via university email, Twitter and subsequent snowballing. Data collection was conducted over the month of June 2022. Questions were presented to participants using the online statistical survey platform LimeSurvey, an open source application.

#### 2.1.2 Cyclist behaviours

Strava is a smartphone app that allows users to record the GPS location and timing of their physical activities, such as running and cycling, and share them publicly or privately on their profile. Strava provides free access to this data to external partners who seek to improve active transport infrastructure. A Research Partnership Request was made to Strava for access to Dublin cycling data. Strava Metro, Strava's data service, aggregates the billions of data points of public cycling activities across street segments, origin and destination polygons which are mapped to OpenStreetMap. Cycling counts are rounded to increments of five, less than three trips on a segment is rounded to zero to protect user privacy. This data is de-identified from Strava users and is provided to research partners in the form of counts of trips along street segments. Street segments within the Dublin city region over the period from May 1st to 31st 2022 were extracted.

#### 2.1.3 Noise

To examine which sources of information could be used to estimate a level of exposure for cyclists, this study considered spatial patterns of noise across the city using predicted noise data from the Environmental Protection Agency (EPA). Dublin City Council's (DCC) strategically placed real-time noise monitors were used to validate the EPA noise maps. These noise maps are made under the framework of the European Environmental Noise Directive [59,60]. The directive has led to increasingly fine resolution noise modelling data for urban agglomerations and further refinement can be expected in the future rounds of noise mapping. Eventually these static noise maps could be replaced with a live data feed through a smart cities approach.

##### 2.1.3.1 EPA strategic noise maps

In global efforts to reduce and protect public health from noise pollution, every 5 years European Member States are legally required to generate strategic noise maps for major roads based on assessments or predictions of noise exposure in the given areas. In Ireland, these maps are created by the National Roads Authority in conjunction with relevant local authorities. Noise levels in decibels (dB(A)) along all roads exceeding 3 million passages per year, are represented in the dataset as polygons of predicted noise contours for L_den_ (day indicator to assess annoyance) and L_night_ (night indicator to assess sleep disturbance). The public can access this data and query noise exposure in areas of interest. The dataset used herein consists of the recent round 3 Road L_den_ released in 2019 obtained from the EPA's geoportal.

##### 2.1.3.2 Dublin City Council strategic noise monitors

**Fig. 3:**
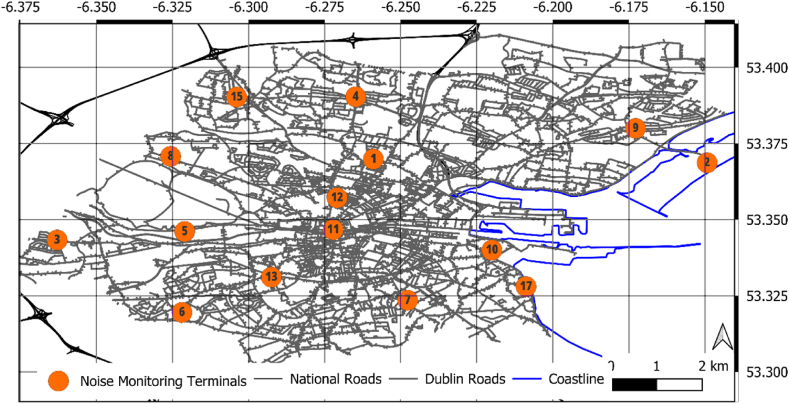
Locations of the 17 noise monitoring stations in the Dublin City Council region.

DCC operates and maintains a network of 24-h environmental sound monitors at 17 locations across the city where quantifying the real-time sound quality is deemed valuable to the community. This network is shown in Fig. 3. Considering that many local authorities lack the resources for extensive data collection campaigns of cyclist noise exposure and this work attempts to demonstrate what can be achieved with existing datasets informed by stationary noise monitors. The ensuing data informs already informs environmental policy decisions and has potential for informing active travel policies. Four noise monitoring stations across the Dublin City region were chosen for their proximity to major urban arterial roads. Sound level data from these stations across the month of May 2022 were obtained from the DCC Dublin City Air and Noise website. The following equation, Eq. (1), was used to convert from hourly averages to L_den_, where *L*_evening_ and *L*_night_ are subject to additional weighting to reflect increased annoyance of noise during evening and night periods:

(1) Lden=10×log10[12×10Lday10+4×10Levening+510+8×10Lnight+101024]

In this case study both the day and evening time periods are relevant for cyclist noise exposure. Due to the fact that L_day/eve_ is not a standard metric it was decided to use the L_den_ value. For the nearest monitoring station to the location used for this case study the L_den_ was 59.8 dBA.

To compare noise exposure of cyclists on direct vs detour routes, Strava segments surrounding these noise monitoring stations were screened for the presence of alternative cycle route options. In other words, Strava records where there was a direct route and a less direct, more often used route for one origin to destination pairing. One suitable segment was found for this analysis in the vicinity of DCC Rowing Club (for location, see Fig. 7a). Along this segment, noise values were extracted and an average equivalent noise exposure for each route was calculated using the following time varying noise equation in Eq. (2):

(2) LAeq,T=10×log10[t1×10L110+t2×10L210+⋯+tn×10Ln10T]

### 2.2 Data analysis

#### 2.2.1 Python

The survey data was processed and analysed in Python (version 3.8.8). Several pre-processing steps were completed before analyses were conducted. Responses to the majority of questions are reported as percentages of the entire sample. Two questions involving categorical answers on a five-point Likert scale required assigning numerical values. Firstly, to calculate the metric for ratings of the soundscape of Dublin, the following coding scheme was applied “Strongly disagree”: 1, “Disagree”: 2 “Neutral”: 3, “Agree”: 4, “Strongly agree”: 5. The negative adjectives (“annoying” and “chaotic”) were reverse coded. Each participant's score across the 5 adjectives were summed and divided by the number of items (5) to produce a soundscape score. Soundscape scores less than 3 were coded as negative, in this way neutrality towards any adjective was considered a positive rating. Secondly, items of the noise sensitivity assessment were similarly assigned numerical scores, with “I get used to most noises without much trouble” reverse coded. These scores were summed and divided by the number of items. Sensitivity scores above 3 were coded as high. Soundscape scores and noise sensitivity scores were then used in statistical analyses.

#### 2.2.2 GIS

To perform a visual analysis of the EPA noise contours and Strava data, data was imported into ArcGIS Pro (Esri Inc., 2020, version 2.9.0). Both datasets were clipped to include only information within the boundaries of the 5 administrative areas of Dublin city [50] and used to create a heat map of total trip counts on each road segment across Dublin. In creating a map of the predicted noise levels across the city, the contour value bands were adjusted for equal subdivisions and to better reflect the WHO health limits of levels greater than 53 dB L_den_ presenting problems to humans [9]. Symbology was changed across visualisations to facilitate a clearer view of insights from the datasets.

Reflecting the evidence of detouring behaviours from the survey, we attempted to estimate the levels of noise exposure across routes selected by cyclists where an alternative more popular route diverges from the most direct route. For this, Strava Dashboard was used to identify segments between one origin and destination in which there was a direct route and a longer, yet more frequently chosen route. The four noise monitoring stations were used as reference points to begin the search process.

## 3 Results

### 3.1 Survey responses

Three-hundred and thirty-five cyclists completed the survey (men = 65%, women 34%, non-binary = 1%). Most respondents had been cycling in Dublin for more than 3 years (75.2%), 54.9% cycle several times a week while 28.4% cycled every day (Table 1). Interestingly, 15.5% stated they had started cycling during the pandemic restrictions. As the dissemination of the survey was in part communicated through university email, Trinity College Dublin's faculty and students could have significantly contributed to the sample. As such, Trinity College Dublin's demographic composition is likely to be more varied than that of a typical corporate entity, and its central location makes it highly accessible to commuters from various locations across the city. For more information regarding the demographic attributes of the sample see Table 1.

**Fig. 4:**
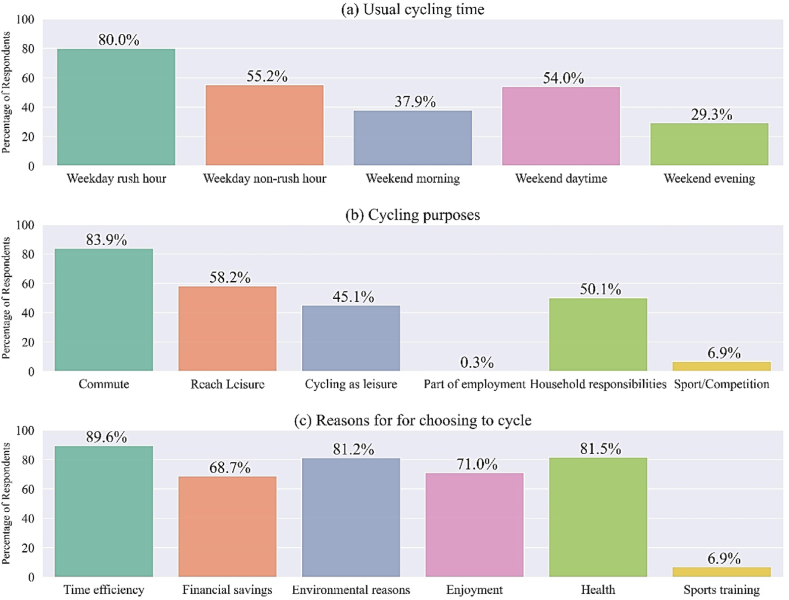
Summary of responses to survey questions enquiring into the time of day usually cycled, purpose, and reasons behind choosing to cycle as their means of transport. Multiple answers were possible.

Weekdays are divided into rush hour and non-rush hour time periods. Due to traditional work patterns weekends in Dublin are not considered to have a rush hour. To avoid confusion in survey respondents the phase rush hour was avoided for the weekends and replaced with morning, daytime and evening periods. The most common time of day to cycle was weekday rush hours (80%), followed by weekday non-rush hours (55.2%) (Fig. 4 a). The ubiquity of cycling during rush hours was reflected in 83.9% of respondents stating a purpose of their cycling was commuting, while approximately half of all respondents acknowledged cycling as a recreational activity itself, to reach leisure, or to engage in household responsibilities, such as shopping or transporting dependents. Only one person stated cycling is part of their employment (Fig. 4 b). When asked about their motivations for choosing to cycle, the majority stated time efficiency (89.6%). This was followed closely by environmental (81.2%) and health reasons (81.5%). Seventy-one percent expressed choosing to cycle because they enjoy it (Fig. 4 c).

In being asked to describe their general experience cycling in Dublin, 66% reported a positive experience, that is, having chosen the descriptor “excellent”, “good”, or “acceptable”. The remaining 34% described their experience as “bad” or “terrible” (Appendix B Fig. B1). However, when asked to appraise the sound environment while cycling, 77% of the sample expressed a negative experience, defined as agreement with negative adjectives and disagreement with positive adjectives (see Appendix A Table A1 for the list of adjectives). Further, descriptions of the soundscape eliciting the highest agreement and disagreement from cyclists were “chaotic” and “calm”, respectively.

Bombardment of the senses can evoke different behavioural coping strategies among cyclists of varying skill levels [61]. In this vein, participants were asked to state whether they engage in specific protective practices to negotiate cycling when they are in loud environments. In terms of behaviours, 30% of the sample said they cycle closer to the kerb, 25% take the centre of the lane, and 22% wear headphones. Regarding psychological reactions, 60% report they are bothered by the noise, 42% actively try to ignore loud noise, 57% feel their body tense, while 47% report getting used to the noise easily. In terms of weighting of different environmental safety considerations, more people worry about air pollution in these contexts than noise: 82% worry about exhaust fume exposure compared to 22% worrying about noise exposure. Interestingly, 7% reported feeling safe in loud environments.

This can exert an emotional toll. Almost half of the sample, 49%, responded that noise has affected their well-being while cycling (Table 2). Closer investigation into specific emotional states revealed a majority of the sample have been left feeling irritable/angry (91%), anxious/nervous (79%), and unhappy (67%) after cycling in Dublin. Further, 32% responded that traffic noise impacts the time of day they choose to cycle (Table 2). While this is a minority, it is noteworthy that it is unlikely that bus or car users would factor noise into their choice of time to travel.

**Table 2:** Percentage of cyclist responses (N = 335) to 4 survey items stating their level of dis/agreement with the influence of traffic noise on their experiences and behaviours.

Survey item	Yes	No
Is the time of day you choose to cycle influenced by traffic noise?	32	68
Is your well-being affected by traffic noise while cycling?	49	51
Do you take any detours to avoid cycling on noisy routes?	39	61

	**Yes**	**No**	**I don't talk to someone while cycling**
When cycling with someone can you hear them talking?	21	35	44

#### 3.1.1 Detouring

For many transport planners and engineers, success in transport is the fastest route from A to B. However, there is compounding evidence that it is not uncommon for cyclists to choose to sacrifice time in return for increased safety or pleasantness [61]. In this sample, 39% answered positively when asked whether they take detours explicitly to avoid noisy routes (Table 2). These detours ranged in length from 4 to 40 min per day, some reported detours amounting to 10–20% extra time. Many participants mentioned choosing paths through parks: *“I'd generally cycle through the Phoenix Park as a detour to avoid traffic build-up and noise, adding* 20 min *to journey”* and *“I take the war memorial gardens route instead, it's more enjoyable”*. One person wrote of the im-permeability of cyclable routes, “*It takes knowing the short cuts though, so I only do this in my locality”* while some mentioned they would like to detour but have few choices available to them, more pleasant, quiet or otherwise “N*o suitable detours available but if I could, I would”.* The presence of children was mentioned as an incentive to detour explicitly to be heard while cycling “*with children, I chose routes with less traffic and less noise, so they can hear me if I'm guiding them*”. Some stated they detour for safety reasons rather than noise pollution *“It's more about avoiding busy routes. Noise is of less concern than being run over”* and *“I always try to go for a safer route but if protected cycle route is noisy, I may still choose it”*. Simultaneously, others reported detouring and recognised a previous lack of consideration for noise “*I hadn't thought about noise in this way before, but it does affect enjoyment and tension”.*

Traditional transport philosophies have ascribed transport to a temporary state, devoid of sociality [61]. However, urban infrastructure use is inherently social, as the life of a city is spoken through what is and is not facilitated [62]. In this regard, participants were asked whether they can hear a companion speak while cycling in Dublin. Forty-four percent stated, *“I don't talk to someone while cycling”* perhaps based on pre-emptive safety concerns due to their close proximity to dense motor traffic. Thirty-five percent responded *‘no’*, that is they may try to cycle with a companion but are physically unable to hear them, while 21% responded *‘yes’* (Table 2).

#### 3.1.2 Noise sensitivity

In general, higher objective noise levels tend to be associated with higher perceived noise levels, but this relationship is not always linear. The relationship between objective and perceived noise levels can be complex and influenced by a range of factors. One hypothesis is that cyclists may experience streets differently according to self-reported noise sensitivity. In this sample, 73% scored as highly noise sensitive, 27% as low. Six statistical tests were conducted at a Bonferroni-corrected α-level of 0.008 to assess the following relationships.

The results of a Mann-Whitney *U* test indicated that ratings of the soundscape do not differ significantly between high (*Md* = 2.4) and low noise sensitive cyclists (*Md* = 2.4, *U* = 11842, *p* = 0.345), we conclude that there is no significant difference between low and high noise sensitive people in their ratings of the Dublin soundscape. Furthermore, five Chi-Square Tests of Independence were performed to assess the relationship between categorical variables, that is, between noise sensitivity and various cycling experiences and behaviours. Firstly, no significant relationship was found between noise sensitivity score and reports of detouring, *X*^*2*^(40, *N* = 335) = 16.33, *p* = 0.999. That is, scoring high in noise sensitivity had no association with reports of taking detours to avoid noisy routes. A second Chi-Square test was conducted revealing no significant relationship between sensitivity score and reporting that noise influences the time of day one chooses to cycle, *X*^*2*^(40, *N* = 335) = 23.14, *p* = 0.985. In an examination of whether high noise sensitivity was related to increased psychological distress, no significant relationship was found, *X*^*2*^(40, *N* = 335) = 25.63, *p* = 0.962. That is, scoring high in noise sensitivity had no association with noise affecting one's wellbeing while cycling. Finally, there was a significant relationship between reports that noise influences one's wellbeing while cycling and both detouring *X*^*2*^(4, *N* = 335) = 16.04, *p* = 0.003 and the time of day one chooses to cycle *X*^*2*^(4, *N* = 335) = 36.36, *p* = 0.000. That is, in feeling distress, people tend to alter their behaviours around the road traffic to mitigate the negative consequences of noise rather than ignoring or persevering through. The importance in behavioural change then appears to be not whether you are more sensitive to noise but whether noise noticeably affects your feelings of wellbeing.

### 3.2 GIS-based mapping

**Fig. 5:**
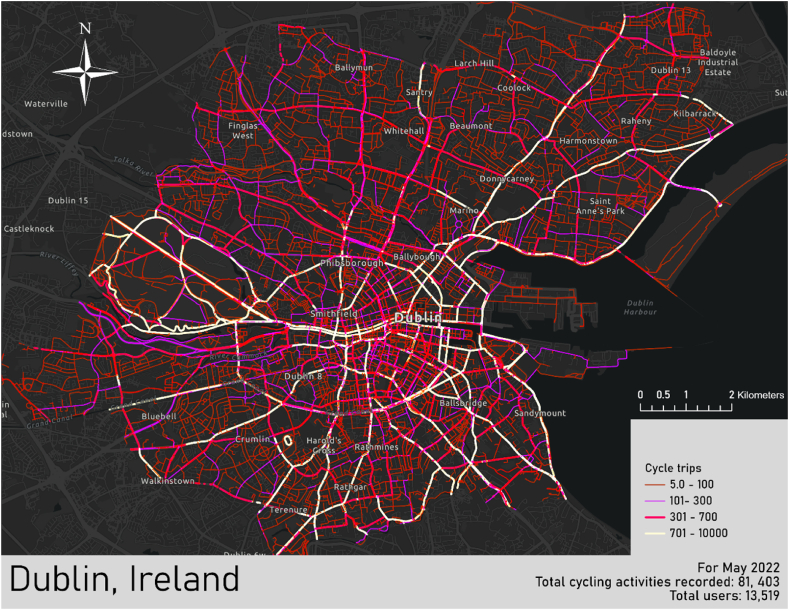
Strava cycling trips across Dublin City administrative areas for the month of May 2022.

This research correlated crowdsourced cycling activities with municipal noise maps through geospatial mapping. Noise and cycling activity were visually assessed individually and compared using a GIS framework to gain further insight into potential patterns and relationships between cyclist route selection and road traffic noise. A total of 81,403 Strava trips were recorded by users in May 2022, with volumes greatest 8am–9am followed by 5pm-6pm. First, a heat map of routes taken by cyclists was created, revealing those most commonly cycled: arterial routes serving the city centre, the banks of the River Liffey, and along the cordon formed by the canals (see Fig. 5). See Table 3 for a brief summary of Strava cycling records for May 2022 and the previous May of 2021. In Table 3, there is a marked difference in the share of commuting and leisure activities between 2022 and 2021, likely reflecting the governmental measures to work from home where possible during the COVID-19 restrictions. The total number of users and activities recorded was lower in 2022, but the average number of trips recorded per user remained similar (6 in 2022 and 5.5 in 2021). Perhaps this reflects a waning popularity of the Strava app, fitness trackers in general, or growing awareness of data privacy concerns.

**Table 3:** Strava cycling trip summary within the Dublin city region for the period May 1st – 31st.

Strava	2022	2021
Users	13,519	17,058
Total activities	81,403	93,801
Commute (%)	40,249 (49%)	30,626 (33%)
Leisure (%)	41,154 (51%)	63,175 (67%)

**Fig. 6:**
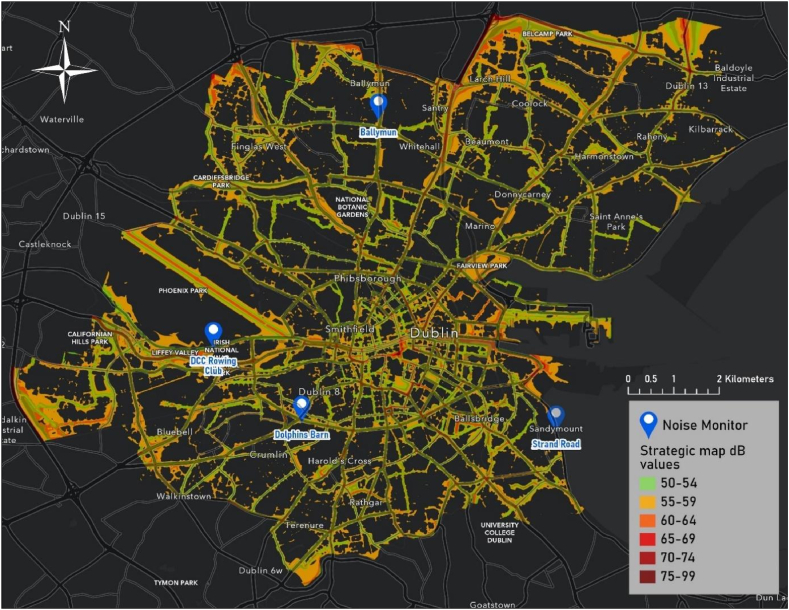
Geospatial map of the 2019 strategic noise data of the Environmental Protection Agency. Colours represent noise contour ranges pertaining to differences in dB(A) levels. Blue place-markers note the locations of noise monitoring stations. (For interpretation of the references to colour in this figure legend, the reader is referred to the Web version of this article.)

Secondly, from the geospatial mapping of EPA noise data across Dublin (Fig. 6) we can see that city noise levels are often predicted to exceed 60 dB (orange to dark red contours), particularly in very central areas and outer routes near the motorway. Over the month of May 2022, the average L_den_ results calculated from the real-time noise monitoring stations were as follows, Dolphins Barn: 62.52 dB, DCC Rowing Club: 59.84 dB, Ballymun: 67.68 dB, and Strand Road: 73.19 dB, somewhat exceeding the strategic map estimations.

**Fig. 7:**
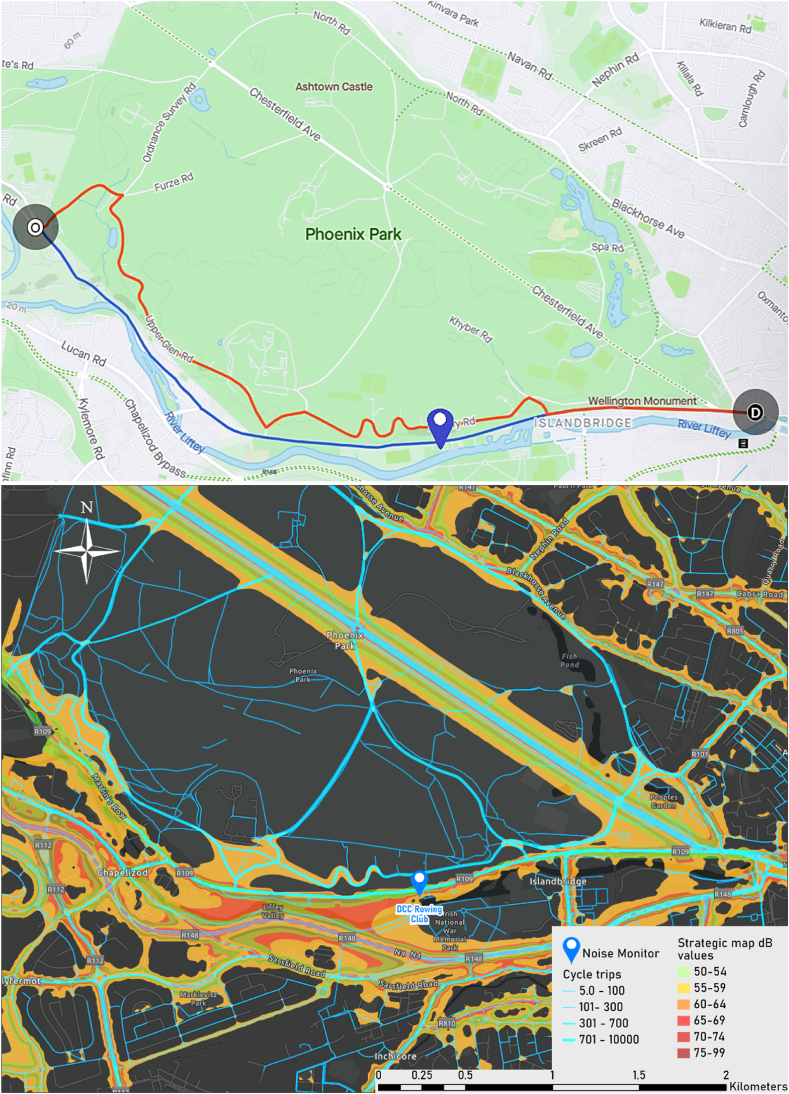
(a). Screenshot of the route on Strava Metro with origin (O) at a Southwest entrance of Phoenix Park at Lower Lucan Road and destination (D) at the Southeast entrance of Phoenix Park at Parkgate Street. The blue line (4.9 km) represents the most direct route, and the red line (5.8 km) shows the most popular route from O to D according to trips logged on Strava. The blue place marker represents the location of the nearest real-time noise monitor, located at the DCC Rowing Club along the banks of the River Liffey. (b) Visualisation of Strava cycling records overlayed with contours of the strategic noise map. The area shown features the direct and popular routes of origin (O) and destination (D) visualised in Fig. 7a. Dark grey regions represent areas for which there was no noise data. The blue marker representing the DCC Rowing Club monitor can be used as a reference. (For interpretation of the references to colour in this figure legend, the reader is referred to the Web version of this article.)

#### 3.2.1 Detour case study

From the Strava trip counts, there was some evidence that cyclists deviate from the most direct routes. This was echoed in the survey responses with 39% stating they detour. Further insights from the survey highlighted that many choose to detour through parks rather than mix with loud traffic. To examine the variations in levels of noise exposure of these detours taken by cyclists, this analysis focused on one route along the south of Phoenix Park near the DCC Rowing Club noise monitor. The origin and destination can be seen in Fig. 7a. The direct segment on the route of focus is a major urban arterial route, with a speed limit of 50 km/h, painted bike lanes on the footpaths, and is featured in blue in Fig. 7a (4.9 km, elevation 17 m). The detour features a shared-use road with a recently updated 30 km/h speed limit through green spaces and forested surroundings, in red in Fig. 7a (5.8 km, elevation 34 m). We overlayed the cyclist records with the EPA noise contours (see Fig. 7b). Values of the noise contours along both the popular and direct route were extracted. The mid-point of each contour band was taken as a proxy to estimate average exposure. For example, 52 dB was taken as the mid-point for the 50–54 dB contour band (see Table 4). As the EPA does not map predicted noise levels below 50 dB, some areas along the routes had no noise values, therefore, a value had to be substituted. The choice of substitute was quite trivial. For ease, 0 dB was chosen. This triviality is outlined in Table 5, where we can see that a choice of 0 dB, 35 dB, or 50 dB makes little difference to exposure levels due to the logarithmic nature of decibels.

**Table 4:** Noise profile for the direct and popular routes consisting of the time spent in each noise contour band when cycling along that route.

Noise contour band (dB(A))	Average level (dB(A))	Time cycling within each noise contour (mins)	
		Direct route	Popular route
0–50	0	0	7.51
50–54	52	1.78	0.15
55–59	57	0	12.06
60–64	62	1.91	0.26
65–69	67	11.78	0
70–74	72	0.43	0
75–99	87	0	0
Total		15.90	19.98

**Table 5:** Average noise exposure calculated for a cyclist on the direct and popular routes showing the difference in exposure level across noise level substitution options for areas where no noise contour data is available.

Routes	Noise level substitution		
	0 dB(A)	35 dB(A)	50 dB(A)
Direct	65.4	65.4	65.4
Popular	55.1	55.1	55.6

**Fig. 8:**
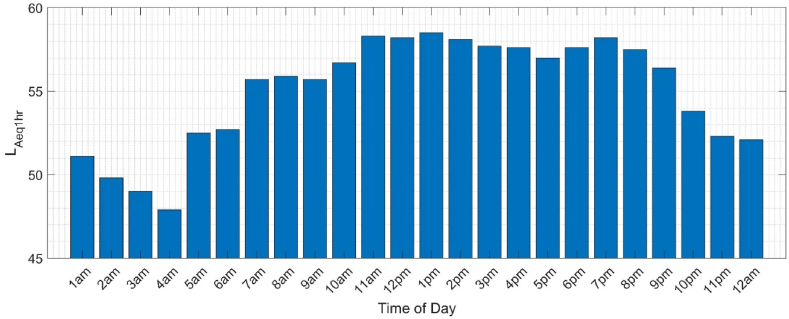
Average LAeq1hr values for the month of May 2022.

The distances of each route and an average urban cycling speed, taken to be 13.5 km/h [63], were used to calculate a noise profile of time exposed to each dB level, for each route (Table 4). To be able to compare exposures for routes of differing lengths, the exposure for a cyclist on an equivalent 20-min journey was calculated using each route's noise profile and Eq. (2). This noise exposure estimation focused on the deviating sub-segment, where the routes deviate to where they re-join, to gauge the different exposure such a detour would equate to for a person. Noise levels were found to be 65.4 dB(A) for the direct route and 55.1 dB(A) for the popular detour route (see Table 5). Therefore, a cyclist's exposure to noise was 10.3 dB(A) higher on the direct route, saving 4 min 5 s compared to someone detouring through the park. A validity check of the nearby real-time noise monitor data was encouraging with an average of 59.8 dB L_den_, the slightly lower level likely reflecting its displaced position from the roadside. The average L_Aeq1hr_ values for the monitoring station during the period of the study are reported in Fig. 8. The above is an estimate of the difference in exposure of cyclists who do and do not take detours. Noise exposure of a cyclist on a journey across the city would differ.

## 4 Discussion

Traditionally, spaces of mobility were considered temporary, transient, and not constitutive of places in which meaning is experienced, a concept owed to non-embodied forms of mobility [61]. However, cycling is embodied, sensorily open to the environment, and socially oriented. The findings herein demonstrate that road traffic noise impacts cyclists negatively. These impacts are often underestimated, with excessive exposure levels potentially detrimental to cyclist's health and well-being as well as sustainable transport goals. The geospatial mapping and route noise-profiling showed that having the option to detour may help individuals avoid negative health consequences of excessive traffic noise exposure. However, insights from the questionnaire revealed many cyclists are not afforded alternative routes. The implications of these findings, related recommendations and policy implications will be highlighted in the following sections.

The majority of this sample reported a positive holistic experience cycling in Dublin but a negative appraisal of the sound environment. In policy and research, urban sounds are frequently taken into account only as psychophysical stressors and, indeed, almost half the sample reported their wellbeing is affected, predominantly being left to feel irritable, angry, anxious, and unhappy after cycling. However, to comprehensively address noise effects, this research drew from soundscape approaches which consider sounds as resources [31]. With the soundscape most frequently described as chaotic and not calm, this speaks to the character impressed on people by the streets of Dublin and is not favourable for a city hoping to increase its cycling modal share from 6 to 13% [49]. As Ingold [64] outlines, the character of a place is ascribable to the *“experiences it affords to those who spend time there to the sights, sounds and indeed smells*”. Local interventions providing alternative urban experiences are needed and are being implemented in comparable European cities, such as low traffic neighbourhoods, car-free centres, and greening strategies [65,66].

The future prospects for the urban noise environment are also influenced by the transition to electric vehicles. Research has shown that there may be a beneficial effect on people's perceptions of traffic noise due to the absence of engine tones and a reduction in noise level from electric vehicles [67,68]. These benefits are potentially offset by an increase in accident risk at low speeds due to the quiet nature of electric vehicles [69]. The methodology proposed in this paper will incorporate this changing road traffic soundscape through regular updates to the END noise maps. The CNOSSOS modelling adopted in EU legislation has open categories for future electric vehicles and is already adapting to the changing noise environment [10,60]. Recent research has demonstrated that the CNOSSOS-EU framework is more accurate than previous methods such as CRTN-TRL [70] and RTN-96 [71]. New data collection strategies, such as the use of UAVs [72], are leading to increased spatial and temporal resolutions for model input data. Such efforts by researchers and policy makers are likely to increase the reliability of strategic noise maps over time. In the interim, this research has demonstrated that the combination of existing datasets can bridge the gap and enable current policy makers to make more informed decisions without the need for high costs data gathering exercises.

It was also found that a majority of these regular cyclists are unable to communicate with a travel companion. This is likely a barrier to enjoyment for many people and points to city inhabitants being let down by the current provision of infrastructure. Being able to communicate appears particularly important for people when cycling with children, for their safety and comfort. Cycling in childhood can influence propensity to cycle in later-life [73] but parental behaviours and attitudes can be very influential, with perception of safety a widely-held priority [74]. To be effective, bicycle infrastructure must prioritise the users’ desires and needs [75], rather than solely considering traffic flow and cost. This lack of provision for communication may have devastating consequences for increasing the uptake of cycling. It has been highlighted how the sociality of cycling is integral to the maintenance of Amsterdam as a “cycling city” in its function of attracting non-Dutch groups with little experience cycling [76]. Further, experiencing a socially engaging area has been shown to compensate for negative influences of high noise pollution [14].

Almost 40% of this sample reported that they take detours (spatial detours) to avoid noisy routes, sacrificing what are often substantial amounts of time for reduced unpleasantness. Additionally, approximately one third of the sample reported that noise influences the time of day they cycle (temporal detours). Traditionally, urban mobility is construed and designed for in terms of rapid connections between origins (A) and destinations (B), defined by the push and pull factors of A and B [61]. However, this research highlights how practitioners must redefine their conceptualisation of urban mobility to incorporate not only push and pull factors, user demand, or network capacity but also the auditory quality of travel experience. Indeed, the Dutch bicycle traffic manual, considered by many to be the gold standard, highlights attractiveness as one of the five crucial components of safe and effective cycle networks, referring to the desirability of one's surrounding environment [77]. Additionally, in terms of successful urban mobility, the temporal and energy efficiency of cycling has been previously acknowledged by municipal decision-makers and cyclists alike. However, similar to this study, cyclists acknowledge more embodied benefits, such as health and enjoyment, than are considered by decision-makers [36], highlighting the need to incorporate user motivations in bicycle network planning and policy.

Annoyance in response to noise is itself associated with increased risk of high blood pressure [78] and cyclists who are highly sensitive to noise have a worse perception of the auditory environment [34]. To avoid reductions in sustainable mobility use, cycling infrastructure must be accessible and comfortable for everyone, not only the most fearless or environmentally insensitive. Our results demonstrated no associations between noise sensitivity and wellbeing, spatial detours, temporal detours, nor ratings of the street soundscape. There was a relationship between reports of noise influencing wellbeing and noise induced temporal and spatial detours. This could suggest that noise noticeably affecting your wellbeing is more significant for behaviour change than a general sensitivity to noise, however, the nature of this relationship was not directly assessed. Ultimately, attempts to mitigate noise impacts would do better to focus on wellbeing in general rather than assigning resources to focus on the experiences of noise sensitive groups.

These results also highlighted that cyclists are bothered by the noise and feel physical and psychological tension but their concerns regarding noise pale in comparison to those regarding physical traffic or traffic fume inhalation, with noise relegated to a “nuisance”. Thus, perceptions of a route as more “pleasant” may implicitly be referring to the absence of bombardment of the senses. This supports both Bickerstaff's [38] finding that the public frequently overlooks or downplays perceptions of environmental threats and earlier findings that the Irish populace gives noise pollution less consideration than is typical of other European citizens [24].

Patterns of cycling can be difficult to monitor, however, ‘big data’ can serve to help researchers and decision-makers make informed estimations. This research visualised historical behaviours of 13,519 people who regularly choose to cycle in a medium city across 81,403 journeys. Despite the local authority having a smart city team supporting mobility projects, current monitoring of cycling modal share in Dublin involves manual counts of people travelling towards the city centre at discrete locations along the canal cordon and on discrete dates each year. These numbers are then used to inform cycling infrastructure developments [52]. However, from our geospatial map, a concentration of cycling within the bounds of the canal cordon is evident. Indeed, the small size, flat gradient, and traffic congestion of Dublin facilitates choosing a bike for short journeys within the city centre. These spatial patterns of cycling attest to the likely underestimation of the City Council's current measurements of cycling demand and, therefore, under-investment in infrastructure provision. Indirect measurements, such as the temporally detailed Strava data, should be taken advantage of to fill existing knowledge gaps. Indeed, using Strava data to gain insight into cycling is becoming increasingly common in research and transport practices [46,79].

In visualising Strava fitness activity, it is also remarkable that social inequities experienced can become discernible on a map. Constructing the Strava heatmap of Baltimore, USA, led to the stark realisation that segregation felt in the streets and opportunities of the city was also apparent in the density of running activities across the city [80]. The cycling map of Dublin shows no such clear-cut inequities, at least when considered in isolation. When integrated with the strategic noise maps and reports of cyclist experiences, we see a picture begin to form of inequity in Dublin. Thirty-nine percent of the sample reported that they take detours, many of whom mentioned this involves routes through parks, gardens, or along the canals. Others reported a desire to avoid traffic but have no detour options. Therefore, integrating multiple datasets can unearth unexpected insights such as the spatially unequal distribution of green routes or public parks. For example, high-income urban areas have been shown to possess more green spaces [81,82] as well as facilitate greater and safer access to cycling provisions [83,84]. Alternatively, a lack of knowledge of alternative routes could speak to a need for more public sharing of area-specific knowledge, for example, hidden trails, and the importance of integrating this within active travel technologies. It should also be noted that research has shown that vegetation is able to improve environmental noise perception which may provide wider benefits [85].

This analysis unexpectedly revealed that the number of users and activities recorded in Dublin declined from 2021 to 2022. This could reflect a waning popularity of the Strava app, or fitness trackers in general, although this seems unlikely as reports attest to the continually growing popularity of self-tracking fitness apps [86]. Alternatively, it may reflect a growing awareness of data privacy concerns, although, it has been noted that while internet users worry about their privacy, they often do not go to the effort of actively determining how they present themselves online [87]. This is mere speculation, however, changing trends in society that might be reflected in changes in technology usage must be investigated if technological tools within smart city initiatives are to serve the interests of citizens. For instance, what public needs are these apps no longer meeting?

As cycling is an activity strongly embodied and highly interactive with the surrounding environment, conditions of the surroundings such as noise pollution exert a much stronger influence on experience than for other transport modes [61]. This analysis examined exposure over a direct and an indirect segment amounting to 65.4 dB(A) and 55.1 dB(A), respectively. These levels are lower but comparable to objective measurements of cyclist noise exposure in larger cities such as Copenhagen, Paris, Montreal, and Ho Chi Minh, which have demonstrated levels between 68.4 dB(A) and 78.8 dB(A) [30,35]. This is an approximate exposure level of one area of Dublin and would differ if estimates were conducted over a greater expanse of the city.

As environmental noise rarely exceeds 70 dB for extended periods, the harmful effects of noise on human health are typically non-auditory [10]. Research has shown that noise exposure in traffic can dominate a person's daily noise exposure [88]. Additionally research has shown that an increase in noise exposure of 5 dB(A) leads to measurable changes in blood pressure and heart rate [89,90]. For every 10 dB L_den_ rise in transportation noise exposure, the risk of Ischaemic Heart Disease is increased by 6% [91]. These increases in daytime noise exposure can also lead to night-time sleep disturbance with only small increases to occupational noise exposure leading to sleep disturbance [92]. Considering a total daily commute of 45 min there can be considerable differences in the L_den_ value for the cyclist due to the traffic noise exposure. For a person who had an otherwise quiet day, with workplace noise exposure of an office [93], the L_den_ calculation can vary from between 2 and 10 dB due to the cycling noise exposures reported here.

In other words, choosing to cycle poses little risk of damage to one's hearing but other risks to health, wellbeing, and society remain significant. Noise levels experienced by cyclists in Dublin are likely having measurable consequences on their health and should inform planning actions to curb noise attributable to motor traffic. Traffic planners must address lowering cyclists' exposure to noise pollution from traffic as well as their risk of collision and other pollutants. Using the methodology outlined here, noise estimation information can be incorporated into local authorities' procedures for infrastructure developments as well as public communication of healthy and pleasant routes. For example, data could be used to inform cyclists of upcoming loud traffic situations and provide information on crowdsourced detours. A similar endeavour is undertaken by the app Waze which provides motorists with real-time updates on situational conditions such road works, accidents, or heavy traffic.

A limitation of this study is the degree of error in the datasets. First, in the interest of user privacy, Strava rounds activities on segments to counts of 5, removing those that have less than 3 trip counts. This was not important for this analysis but is important to note for future fine-grained analyses. Secondly, the road traffic noise contours are estimations by the EPA, therefore exposure calculations herein are estimations. Further, averaging noise levels, while facilitating ease of exposure estimation, operates within the implicit assumption of a stationary nature of exposure [94]. Finally, there is no temporal overlap between the time spans covered by the Strava data and the noise maps, this temporal misalignment can be mitigated by considering the noise monitoring station. The noise monitoring station data can be aligned with the time period of the Strava data and can be used as a check on the validity of the noise mapping data for that period.

In addition, previous qualitative research has noted the utility of traffic noise during cycling to predict when there is a need to be more vigilant or defensive [61]. This advantage of noise was not included in the survey nor was it mentioned by the participants. This may suggest a degree of priming in the questions towards regarding only the negative consequences of noise, however, 7% of the sample did report feeling safe in loud environments. A comment section on this question could have provided the opportunity for deeper insight into participants responses. Further, in asking participants about their wellbeing while cycling, the concept was not defined and was only assessed via one question. Therefore, it may have had different implications for different people, for example, considering it in more hedonist or eudemonist terms. Previous studies have proposed multifaceted measurements [95]. However, for the purposes of this research, participant's conception of the term was deemed less important than assessing whether participants felt impacted by noise. Additionally, due to time constraints, this study extracted data from Strava and the noise monitors for only one month, May 2022. Further work should consider a seasonal analysis as both cycling patterns and noise levels may vary throughout the year with changes in weather and daylight availability [96]. Finally, future research could filter subsets of the Strava data, such as by trip purpose or demographics [79], to investigate whether specific cohorts show more behavioural aversion to high traffic routes in efforts to inform more equitable transport policy and planning.

## 5 Conclusion

This study has demonstrated a mixed-method approach integrating multiple datasets to estimate urban cyclist noise exposure and gain insight into the influences of traffic noise on cyclists' experiences and behaviours. The intention of this study was to demonstrate the utility of existing data sets to provide evidence for informed policy decisions. It was demonstrated that the weakness of the noise mapping data set could be partially offset by the noise monitoring stations. The noise map is spatially detailed data, and the noise monitoring stations are temporally detailed data. In combination these datasets can provide a valid estimate of noise levels experienced by cyclists. This works shows that useful conclusions can be drawn for active travel planning from noise mapping data and in future policy makers could integrate active travel planning into their noise action plans under noise mapping frameworks, such as the EU's Environmental Noise Directive.

It is important to consider the detrimental health effects of noise exposure in the planning of future cycling infrastructure. Spatial visualisations of exposure data and cycling activity using geographic information system (GIS) approaches can be used in communications with and by decision-makers. Using this method, this study provided evidence that cyclists are detouring and in doing so are reducing their exposure to noise and, therefore, noise-induced health risks. Transport practitioners must adopt practices that include noise exposure to reduce the time and costs associated with preventative detouring cyclists engage in to mitigate stressful situations. This study has also highlighted the value of direct cyclist experiences in understanding individual cycling behaviours. As cyclists are not one homogenous group, this may be crucial to encourage cycling and address the concerns of cyclists at greatest risk of being perturbed by high noise levels. Ultimately, this study has shown that crowdsourcing for exposure estimation is feasible and therefore can be automated and scaled-up for use within local governments to inform cycling developments.

## Funding

This research did not receive any specific grant from funding agencies in the public, commercial, or not-for-profit sectors.

## Ethics statement

All researchers at Trinity College Dublin are required to conduct their research to the highest ethical standards and each individual researcher is responsible for ensuring good ethical practice. The School of Engineering Research Ethics Committee (REC) reviewed the work within this project as part of the MSc dissertation of Rebecca Wogan.

## Data availability statement

Data will be made available on request.

## CRediT authorship contribution statement

**Rebecca Wogan:** Writing – review & editing, Writing – original draft, Visualization, Validation, Software, Resources, Methodology, Investigation, Formal analysis, Data curation, Conceptualization. **John Kennedy:** Writing – review & editing, Supervision, Resources, Funding acquisition, Formal analysis, Data curation, Conceptualization.

## Declaration of competing interest

The authors declare that they have no known competing financial interests or personal relationships that could have appeared to influence the work reported in this paper.
