# Supplementary material for: Crowdsourced cycling data applications to estimate noise pollution exposure during urban cycling
Source: Heliyon. 2024 Mar 17;10(6):e27918. doi: 10.1016/j.heliyon.2024.e27918 (PMC10963327; doi:10.1016/j.heliyon.2024.e27918)
Supplement: Multimedia component 1 [file mmc1.pdf]

Supplementary Material

Appendix A

Table 1. Outline of the categories of the questions, survey questions and answer options. Categories were not displayed to participants.

Question Category	Question	Answer Options
Nature and level of cycling experience	How long have you been cycling in Dublin?	Less than 3 months / 3 months – 1 year / 1-3 years / +3 years
	Did you start cycling during the pandemic restriction period?	Yes / No
	How often do you currently cycle in Dublin?	Every day / Several times a week / About once a week / Several times a month / Less than once a month
	At what time do you usually cycle? (Check all that apply)	Weekday rush hour /Weekday non-rush hour / Weekend morning / Weekend daytime / Weekend evening
	What is the general purpose of your cycling in Dublin? (Check all that apply)	Work or school commute / To reach leisure activities / Cycling as leisure activity itself / It's part of your employment e.g., food delivery / Household responsibilities

		e.g., shopping or bringing children to activities / Sport or competition
	Why do you choose to cycle as your form of transportation? (Check all that apply)	Time efficiency / Cost savings / Environmental factors / Enjoyment / Health or fitness / Extra training for sport cycling
Cycling experience	How would you generally describe your experience cycling in Dublin?	Excellent / Good / Acceptable / Bad / Terrible
	Do you have a favourite time of day to cycle? Please comment on when and why.	Yes / No / Comment
	Overall, the sound environment when cycling in Dublin is: Vibrant/Calm/Pleasant/Chaotic/Annoying.	Strongly Agree – Strongly Disagree (1-5)
	Do you think the time of day you choose to cycle is influenced by the level of road traffic noise?	Yes / No
	Do you think your general well-being has been affected in some way by road traffic noise while cycling?	Yes / No
Behaviours	When cycling with someone else can you hear them talking?	Yes / No / I don't talk with someone while cycling
	When cycling in a loud environment:<br>I am bothered by the noise / I move to cycle closer to the kerb/I actively try to ignore the noise / I enjoy myself / I move to cycle away from the kerb and towards the centre of the lane / I get used to the noise without much trouble / I worry about being exposed to exhaust fumes/ I feel safe/I feel parts of my body tense up / I wear headphones / I worry about noise exposure.	Strongly Agree – Strongly Disagree (1-5)

	Do you take any detours to avoid cycling on noisy routes? Please comment estimating how much time per week you add to your journeys via these detours.	Yes / No / Comment
	Has cycling in Dublin ever left you feeling: Irritable/Very tired/Unhappy/Anxious/Nervous/Unsociable/Headaches, upset stomach?	Often / Rarely / Unsure / Never
Noise sensitivity	Considering your life in general, please state to what extent you agree with each of the following statements: I wake up quickly because of noise/ I am bothered when my neighbours are noisy / I get used to most noises without much trouble / I am sensitive to noise/ Sometimes noise makes me nervous / Music that I usually love bothers me when I am trying to focus / I find it difficult to relax in a noisy place / It does not matter what's happening around me, I can always concentrate well / I get angry with people making noise preventing me from sleeping or working.	Completely Agree – Completely Disagree (1-5)
Demographics	What age are you?	18-25 / 26-35 / 36-45 / 46-55 / 56-65 / 65+
	Which gender do you most identify with? (If you prefer to self-describe, please leave a comment).	Woman / Man / Transgender woman / Transgender man / Non-binary / Prefer to self-describe / Prefer not to say
	Which best describes your current life situation?	Student / Employed / Unemployed / Non-waged work e.g., domestic care

Strava Access Permission	This is not a mandatory question and is completely voluntary.<br>If you use the STRAVA app to record your cycling activities, do you give permission to the researcher, Rebecca Wogan, to follow you on STRAVA and access the cycle route information you have publicly viewable? This data will be accessed solely by the primary researcher, Rebecca Wogan. Once collected, the data will be anonymised, aggregated with other participants' data, and used to conduct analyses on the use of specific cycling routes of interest in Dublin.<br>If you consent to this, please provide your STRAVA username in the comment section.	Yes, I give my permission / No answer

Appendix B

Survey and sound analyses available to view on GitHub in the following repository:

https://github.com/rebwog/Cycling_noise
