# Supplementary material for: Crowdsourced cycling data applications to estimate noise pollution exposure during urban cycling
Source: Heliyon. 2024 Mar 17;10(6):e27918. doi: 10.1016/j.heliyon.2024.e27918 (PMC10963327; doi:10.1016/j.heliyon.2024.e27918)
Supplement: Multimedia component 2 [file mmc2.docx]

**Appendices**

**Appendix A**

**Table A.1** Outline of the categories of the questions, survey questions and answer options. Categories were not displayed to participants.

| **Question Category** | **Question** | **Answer Options** |
| --- | --- | --- |
| Nature and level of cycling experience | How long have you been cycling in Dublin? | Less than 3 months / 3 months – 1 year / 1-3 years / +3 years |
|  | Did you start cycling during the pandemic restriction period? | Yes / No |
|  | How often do you currently cycle in Dublin? | Every day / Several times a week / About once a week / Several times a month / Less than once a month |
|  | At what time do you usually cycle? (Check all that apply) | Weekday rush hour /Weekday non-rush hour / Weekend morning / Weekend daytime / Weekend evening |
|  | What is the general purpose of your cycling in Dublin? (Check all that apply) | Work or school commute / To reach leisure activities / Cycling as leisure activity itself / It’s part of your employment e.g., food delivery / Household responsibilities e.g., shopping or bringing children to activities / Sport or competition |
|  | Why do you choose to cycle as your form of transportation? (Check all that apply) | Time efficiency / Cost savings / Environmental factors / Enjoyment / Health or fitness / Extra training for sport cycling |
| Cycling experience | How would you generally describe your experience cycling in Dublin? | Excellent / Good / Acceptable / Bad / Terrible |
|  | Do you have a favourite time of day to cycle? Please comment on when and why. | Yes / No / Comment |
|  | Overall, the sound environment when cycling in Dublin is: Vibrant/Calm/Pleasant/Chaotic/Annoying. | Strongly Agree – Strongly Disagree (1-5) |
|  | Do you think the time of day you choose to cycle is influenced by the level of road traffic noise? | Yes / No |
|  | Do you think your general well-being has been affected in some way by road traffic noise while cycling? | Yes / No |
| Behaviours | When cycling with someone else can you hear them talking? | Yes / No / I don’t talk with someone while cycling |
|  | When cycling in a loud environment:  I am bothered by the noise / I move to cycle closer to the kerb/I actively try to ignore the noise / I enjoy myself / I move to cycle away from the kerb and towards the centre of the lane / I get used to the noise without much trouble / I worry about being exposed to exhaust fumes/ I feel safe/I feel parts of my body tense up / I wear headphones / I worry about noise exposure. | Strongly Agree – Strongly Disagree (1-5) |
|  | Do you take any detours to avoid cycling on noisy routes? Please comment estimating how much time per week you add to your journeys via these detours. | Yes / No / Comment |
|  | Has cycling in Dublin ever left you feeling: Irritable/Very tired/Unhappy/Anxious/Nervous/Unsociable/Headaches, upset stomach? | Often / Rarely / Unsure / Never |
| Noise sensitivity | Considering your life in general, please state to what extent you agree with each of the following statements:  I wake up quickly because of noise/ I am bothered when my neighbours are noisy / I get used to most noises without much trouble / I am sensitive to noise/ Sometimes noise makes me nervous / Music that I usually love bothers me when I am trying to focus / I find it difficult to relax in a noisy place / It does not matter what's happening around me, I can always concentrate well / I get angry with people making noise preventing me from sleeping or working. | Completely Agree – Completely Disagree (1-5) |
| Demographics | What age are you? | 18-25 / 26-35 / 36-45 / 46-55 / 56-65 / 65+ |
|  | Which gender do you most identify with? (If you prefer to self-describe, please leave a comment). | Woman / Man / Transgender woman / Transgender man / Non-binary / Prefer to self-describe / Prefer not to say |
|  | Which best describes your current life situation? | Student / Employed / Unemployed / Non-waged work e.g., domestic care |

**Appendix B**


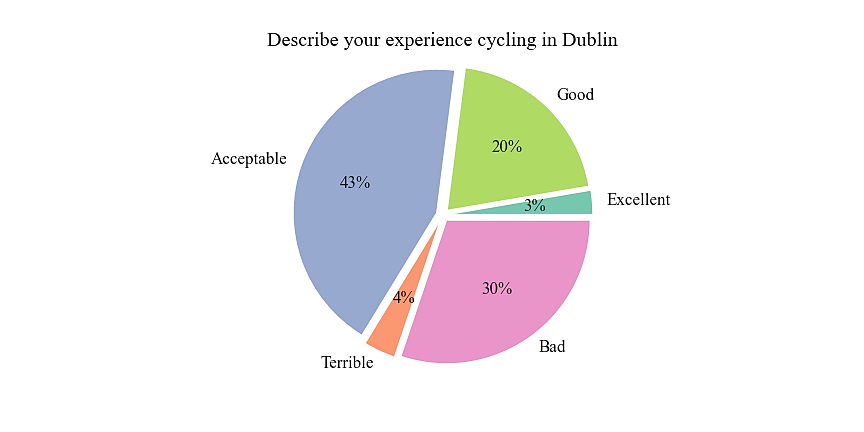


**Fig. B.1** The percentage of survey respondents in agreement with adjectives to categorise their general experience of cycling in Dublin.
